# A classical regression framework for mediation analysis: fitting one model to estimate mediation effects

**DOI:** 10.1093/biostatistics/kxx054

**Published:** 2017-10-26

**Authors:** Christina T Saunders, Jeffrey D Blume

**Affiliations:** Department of Biostatistics, Vanderbilt University, West End Ste., Nashville, TN, USA

**Keywords:** Direct and indirect effect, Intermediate variable, Mediation analysis, Multiple mediators, Regression

## Abstract

Mediation analysis explores the degree to which an exposure’s effect on an outcome is diverted through a mediating variable. We describe a classical regression framework for conducting mediation analyses in which estimates of causal mediation effects and their variance are obtained from the fit of a single regression model. The vector of changes in exposure pathway coefficients, which we named the essential mediation components (EMCs), is used to estimate standard causal mediation effects. Because these effects are often simple functions of the EMCs, an analytical expression for their model-based variance follows directly. Given this formula, it is instructive to revisit the performance of routinely used variance approximations (e.g., delta method and resampling methods). Requiring the fit of only one model reduces the computation time required for complex mediation analyses and permits the use of a rich suite of regression tools that are not easily implemented on a system of three equations, as would be required in the Baron–Kenny framework. Using data from the BRAIN-ICU study, we provide examples to illustrate the advantages of this framework and compare it with the existing approaches.

## 1. Introduction

Mediators are behavioral, biological, psychological, or social constructs that transmit the effect of one variable to another. Mediation analysis seeks to understand how much of an exposure’s effect on an outcome is diverted through a mediating variable (Woodworth, 1928; Alwin and Hauser, 1975; Baron and Kenny, 1986). Background information on mediation analysis can be found in Baron and Kenny (1986), MacKinnon (2008), Hayes (2013), Preacher (2015), and VanderWeele (2015). Modern scientific investigations, such as genetic pathway analysis and disease prevention research, require a sophisticated framework for conducting mediation analysis.

The literature on mediation analysis is largely comprised of the approach popularized by Baron and Kenny (1986), the causal inference framework (Robins and Greenland, 1992; Pearl, 2001; Imai *and others*, 2010; VanderWeele, 2015), and the structural equation modeling approach Gunzler *and others*, 2013. Having been cited over 70 000 times (Google Scholar), the Baron–Kenny causal steps approach is ubiquitous in the social sciences and considered to be a cornerstone of mediation analysis. However, a growing technical literature has pointed out its inability to handle complex mediation hypotheses (MacKinnon *and others*, 2002; Fritz and MacKinnon, 2007; Preacher and Hayes, 2008; Zhao *and others*, 2010; Hayes, 2013).

Here, we propose a classical regression framework for conducting mediation analysis with linear models. We introduce the essential mediation components (EMCs), a general form for the difference in the exposure pathway coefficients. A formula for the EMCs and their model-based variance are derived from the fit of a single well-specified regression model. For the simple mediation model, the indirect effect for a unit change in the exposure is mathematically equivalent to the EMC; in general, however, causal mediation estimands (e.g., portion eliminated [PE] and natural indirect effect [NIE]) and their variance are *functions* of the EMCs, a critical distinction. A closed-form expression for the variance is a welcome advance of the framework, eliminating the need for delta method or resampling approximations.

This approach extends to settings with multiple mediators, interactions, and nonlinearities. Fitting a single model allows for a clean application of regression tools (e.g., imputation of missing data, cross-validation, and penalized likelihood methods) that are not easily implemented in a system of three equations. In a series of examples using data from the BRAIN-ICU study (Pandharipande *and others*, 2013), we illustrate our method and compare it with the existing regression-based approaches. Note that for space considerations, this article focuses on the setting of continuous outcomes (i.e., linear models). Extensions to the generalized linear model framework are in progress.

## 2. Background and notation

### 2.1. The simple mediation model

**Fig. 1.**
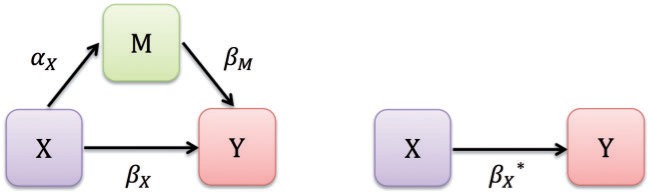
Simple mediation model for exposure $X$, continuous mediator $M$, and continuous outcome $Y$. The coefficients $\alpha_X,\beta_X,\beta_M,$ and $\beta_X^*$ are estimated from the system of three regression equations.

Mediation analyses generally seek to partition the *total effect* of an exposure into its *direct* and *indirect* components. For exposure $X$, continuous mediator $M$, and continuous outcome $Y$, the classic Baron–Kenny *simple mediation model* is illustrated in Figure 1 and represented by the following three regression equations. Errors are assumed to be normally distributed.

(2.1) \begin{align*} \text{E}[Y|X,M] &= \beta_0 + \beta_X X + \beta_M M, \label{eq1}\\ \end{align*}

(2.2) \begin{align*} \text{E}[M|X] &= \alpha_0 + \alpha_X X, \label{eq2}\\ \end{align*}

(2.3) \begin{align*} \text{E}[Y|X] &= \beta_0^* + \beta_X^* X. \label{eq3} \end{align*}

**Fig. 2.**
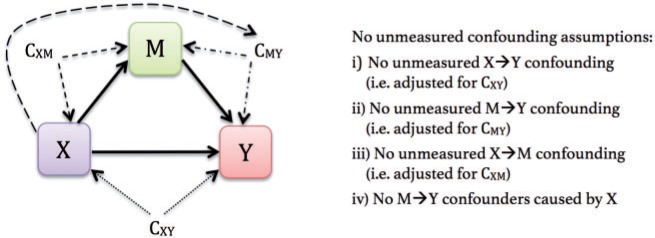
Assumptions required for estimating causal mediation effects from a model with exposure $X$, mediator $M$, and outcome $Y$. $C_{XY}$ represents confounding variables of the $X\rightarrow Y$ relationship, $C_{MY}$ represents confounders of the $M\rightarrow Y$ relationship, and $C_{XM}$ represents confounders of the $X\rightarrow M$ relationship. To estimate causal mediation effects, the researcher assumes that he has controlled for $C_{XY}, C_{MY}, C_{XM}$ and that there are no M$\rightarrow$Y confounders caused by $X$. Identifying CDEs requires that assumptions (i) and (ii) be met. Identifying NDEs and NIEs requires that all four assumptions be met. Figure adapted from VanderWeele’s 2015 book *Explanation in Causal Inference: Methods for Mediation and Interaction*.

The estimated total and direct effects for a unit change in $X$ are $\hat{\beta}_X^*$ and $\hat{\beta}_X$, respectively. The indirect effect of $X$ is commonly estimated using the difference of coefficients, $\hat{\beta}_X^* - \hat{\beta}_X$, or the product of coefficients, $\hat{\alpha}_X \hat{\beta}_M$. For the simple mediation model, the two approaches agree and the total effect of $X$ on $Y$ is the sum of the direct and indirect effects: $\hat{\beta}_X^* = \hat{\beta}_X + \hat{\alpha}_X \hat{\beta}_M$. To infer causality, one must assume the relevant confounders (enumerated in Figure 2) have been accounted for (VanderWeele, 2015). Although the original Baron–Kenny model did not include covariates, we emphasize the importance of adjusting for confounders of the type listed in Figure 2, so that mediation effects are identifiable. One can simply add the set of relevant confounders to each model. Furthermore, the simple mediation model assumes linear relationships and no interaction among the variables, an assumption that can be relaxed. As in any scientific investigation, the assumed causal relationships in a mediation model rely on theory and empirical evidence.

### 2.2. The difference of coefficients approach

There is disagreement as to whether the difference or product of coefficients approach is preferable (Alwin and Hauser, 1975; Preacher and Hayes, 2008; Imai *and others*, 2010). Although the two approaches agree for linear models, in general, they “represent legitimate intuitions in pursuit of two distinct causal quantities” and are not equivalent (Pearl, 2012b). In Section 3.1, we provide a general formula for the difference of coefficients approach (i.e., the PE), which seeks to evaluate the reduction in the total effect if indirect paths were blocked. This approach is recognized as being of great importance in public health policy research (Pearl, 2012a; VanderWeele, 2013; Naimi *and others*, 2014; VanderWeele, 2015). For example, when studying how an intervention can prevent adverse health outcomes, the difference of coefficients measures the maximum preventive effect of any such intervention on the mediating pathways (Pearl, 2012a; VanderWeele, 2015). Relevant quotes concerning the difference of coefficients approach and the PE are included in Appendix A of supplementary material available at *Biostatistics* online.

### 2.3. The causal inference framework for mediation analysis

The causal inference framework for mediation analysis defines mediation effects as contrasts in average potential outcomes (Holland, 1986; Robins and Greenland, 1992; Pearl, 2001). Let $Y_{xm}$ be the potential outcome that would be observed if the exposure $X$ were equal to $x$ and the mediator $M$ were equal to $m$. Let $Y_{x M_{x^*}}$ be the potential outcome that would be observed if the exposure were equal to $x$, but the mediator $M$ were equal to the value it would have been if the exposure was equal to $x^*$. Note that the counterfactuals $Y_{x M_{x^*}}$ and $Y_{x^* M_{x}}$ can never be observed. Table 1 provides the counterfactual definitions of causal mediation effects. We use the $(x,x^*)$ notation to make explicit that causal mediation effects are defined for any two levels of the exposure. When $X$ is a binary variable, the only possible pair of values is $(0,1)$.

**Table 1.** Counterfactual definitions of causal mediation effects and the corresponding regression-based estimands obtained from the simple mediation model

Causal mediation effects		The simple mediation model
Causal effect	Potential outcome	Regression estimand
CDE($x,x^*,m$)	$\text{E}[Y_{xm} - Y_{x^*,m}]$	$\beta_X(x-x^*)$
NDE($x,x^*$)	$\text{E}[Y_{xM_{x^*}} - Y_{x^*M_{x^*}}]$	$\beta_X(x-x^*)$
TE($x,x^*$)	$\text{E}[Y_{x} - Y_{x^*}]$	$\beta_X^*(x-x^*)$
NIE($x,x^*$)	$\text{E}[Y_{xM_x} - Y_{xM_{x^*}}]$	$(\beta_X^* - \beta_X)(x-x^*)$
PE($x,x^*$)	$\text{E}[Y_{x} - Y_{x^*} - (Y_{xm} - Y_{x^*m})]$	$(\beta_X^* - \beta_X)(x-x^*)$

The causal mediation literature distinguishes between *controlled* and *natural* effects. The controlled direct effect (CDE) measures the effect of $X$ on $Y$ while holding the mediator fixed at level $m$ for everyone in the population. The natural direct effect (NDE) measures the effect of the exposure on the outcome when each individual’s mediator is fixed to $M_{x^*}$, what it would have been “naturally” had the exposure been absent (or equal to some referent value). The NIE represents the difference in the outcome if one holds the exposure at level $x$ and changes the mediator from the value that would have been observed under the referent exposure, $M_{x^*}$, to the value that would have been observed under treatment, $M_x$. The NIE is the difference between the total effect and the NDE: $\text{NIE} = \text{TE} - \text{NDE}$. Another important quantity is the PE, which is the difference between the total effect and the CDE: $\text{PE} = \text{TE} - \text{CDE}$ (VanderWeele, 2015).

Pearl’s *mediation formula* (see Appendix B of supplementary material available at *Biostatistics* online) is a generalization of the product of coefficients approach and can be used to estimate the causal mediation effects from any type of model (Pearl, 2001; Imai *and others*, 2010; Pearl, 2012a). For illustrative purposes, Table 1 displays the regression estimand obtained from applying the mediation formula to the simple mediation model: $\text{TE} = \beta_X^*(x-x^*),$$\text{NDE} = \beta_X (x-x^*) = \text{CDE}$, and $\text{NIE} = \alpha_X \beta_M (x-x^*) =$ PE. In this simple case, the estimated effects are identical to those obtained in the Baron–Kenny approach for a unit change in the exposure.

### 2.4. Estimating the variance of mediation effects

Currently, estimating the variance of the indirect effect relies on approximations; a closed-form solution has not been discovered until now. Sobel’s (1982) delta method approximation for the variance of the product of coefficients is $\widehat{\text{Var}}(\hat{\alpha}_X \hat{\beta}_M) = \hat{\alpha}_X^2 s_{\beta_M}^2 + \hat{\beta}_M^2 s_{\alpha_X}^2$. Even though the sampling distribution of $\hat{\alpha}_X \hat{\beta}_M$ tends to be skewed and highly leptokurtic, inference procedures rely on a large sample normal approximation; as a result, Sobel confidence intervals (CIs) tend to lie to the left of the true value for positive indirect effects and to the right for negative indirect effects (Stone and Sobel, 1990; MacKinnon *and others*, 1995, 2004). VanderWeele has derived delta method variance approximations for more complex mediation models (2015). Bootstrapping handles asymmetric sampling distributions better than the delta method and thus improves the accuracy of confidence limits (Preacher and Hayes, 2008). Monte Carlo methods estimate the variance by simulating the sampling distribution of mediation effects (MacKinnon *and others*, 2004) and are implemented in the software by Imai *and others* (2010). Now that an analytical solution for the variance exists, it is of interest to re-examine the behavior of these approximations. Simulations in Section 5.2 shed light on these considerations and the efficiency gains inherent in avoiding conservative approximations.

## 3. A classical regression framework

We define an intermediate inferential target called the EMCs, which is the vector of changes in the exposure coefficients. Analytical estimates of the EMCs and their model-based variance are derived from the fit of a single regression model. Inference for causal mediation effects, which are functions of the EMCs, follows naturally. Furthermore, because the fit of only one model is required, it is straightforward to incorporate multiple mediators, exposure–exposure interactions, and mediator–mediator interactions.

### 3.1. The essential mediation components

Recall that the simple mediation model (2.1–2.3) assumes $X$ is linearly related to $Y$. A more general formulation allows the effect of $X$ to be nonlinear: $\text{E}[Y|X,M] = \beta_0 + \beta_X h(X) + \beta_M M$, where $h(X)$ is a flexible function of $X$ (e.g., $log(X)$). For $p$ exposures $\boldsymbol{X}$ and $j$ mediators $\boldsymbol{M}$, the full model and its implied submodel are

(3.1) \begin{align*} \label{eq:full_model} \text{E}[Y|\boldsymbol{X},\boldsymbol{M}] &= \beta_0 + \boldsymbol{h(X)}\boldsymbol{\beta_X} + \boldsymbol{M}\boldsymbol{\beta_M}, \\ \end{align*}

(3.2) \begin{align*} \label{eq:sub_model} \text{E}[Y|\boldsymbol{X}] &= \beta_0^* + \boldsymbol{h(X)} \boldsymbol{\beta_X^*}, \end{align*}

where $\boldsymbol{h}(\boldsymbol{X})$ is a vector that captures the nonlinear trends in $\boldsymbol{X}$, such as a spline basis. We call the vector of differences $\Delta = \boldsymbol{\beta_X^*} - \boldsymbol{\beta_X}$ the EMCs.

Using properties of the multivariate Gaussian distribution, we obtain estimates of the EMCs and their variance using functionals from the fitted full model (3.1). Under well-known conditions on the linear model, $\sqrt{n}(\boldsymbol{\hat{\beta}}- \boldsymbol{\beta}) \sim \text{MVN}_k(0,\boldsymbol{\Sigma})$, where $k = p+j$ is the number of parameters in the full model. Without loss of generality, we consider a model with no intercept. Partition $\boldsymbol{\hat{\beta}} = (\boldsymbol{\hat{\beta}_X},\boldsymbol{\hat{\beta}_M})'$, where $\boldsymbol{\hat{\beta}_X}$ is the $p$-vector of exposure coefficients and $\boldsymbol{\hat{\beta}_M}$ is the $j$-vector of mediator coefficients such that

\begin{equation*}
\left[ {\matrix{\boldsymbol{\hat{\beta}_X} \cr \boldsymbol{\hat{\beta}_M} \cr }}\right] \sim \text{MVN}_k \left(\left[ {\matrix{\boldsymbol{\beta_X} \cr \boldsymbol{\beta_M} \cr }}\right], \left[ {\matrix{\boldsymbol{V_{X}} & \boldsymbol{V_{XM}} \cr \boldsymbol{V_{MX}} & \boldsymbol{V_{M}} \cr }}\right] \right)\!. \end{equation*}

The conditional distribution of $\boldsymbol{\hat{\beta}_X}$ given $\boldsymbol{\hat{\beta}_M} = \boldsymbol{b_M}$ is $(\boldsymbol{\hat{\beta}_X}|\boldsymbol{\hat{\beta}_M}=\boldsymbol{b_M}) \sim \text{MVN}_p(\boldsymbol{\beta_{X|M}},\boldsymbol{V_{X|M}})$, where $\boldsymbol{\beta_{X|M}} = \boldsymbol{\beta_X} + \boldsymbol{V_{XM}}\boldsymbol{V_{M}^{-1}}(\boldsymbol{b_M} - \boldsymbol{\beta_M})$ and $\boldsymbol{V_{X|M}} = \boldsymbol{V_{X}} - \boldsymbol{V_{XM}}\boldsymbol{V_{M}^{-1}}\boldsymbol{V_{MX}}$. If $\boldsymbol{b_M}=0$, we obtain $(\boldsymbol{\hat{\beta}_X}|\boldsymbol{\hat{\beta}_M}=0) \sim \text{MVN}_p(\boldsymbol{\beta_X^*},\boldsymbol{V_X^*})$, where $\boldsymbol{\beta_X^*} = \boldsymbol{\beta_X} - \boldsymbol{V_{XM}}\boldsymbol{V^{-1}_{M}}\boldsymbol{\beta_M}$. Thus, a general formula for the difference in pathway coefficients $\boldsymbol{\beta_X^*} - \boldsymbol{\beta_X}$, which we call the EMCs of $\boldsymbol{X}$, is

(3.3) \begin{equation*} \label{eq:EMCformula} \Delta = \boldsymbol{\beta_X^*} - \boldsymbol{\beta_X} \equiv -\boldsymbol{V_{XM}}\boldsymbol{V_{M}^{-1}}\boldsymbol{\beta_M}. \end{equation*}

This formula allows us to estimate multidimensional mediation effects ($\boldsymbol{X}$ and $\boldsymbol{M}$ can be multivariate) from a single regression model (3.1) rather than fitting separate models and aggregating effect estimates. Notice that for the simple mediation model (2.1–2.3), $\Delta = \beta_X^* - \beta_X$ is exactly equal to the PE for a unit change in $X$ (which equals the causal NIE and the Baron–Kenny product of coefficients estimand). In general, when the exposure or mediator effects are nonscalar, the PE is a function of $\Delta$:

(3.4) \begin{equation*} \label{eq:PE} \text{PE}(x,x^*) = [\boldsymbol{h}(x)-\boldsymbol{h}(x^*)] \Delta. \end{equation*}

Table 2 of supplementary material available at *Biostatistics* online provides a list of commonly encountered mediation models for which the controlled direct effects and natural direct effects are equivalent, and as a result, the PE and the NIE are the same. For these models, the NIE can be estimated using our formula: NIE($x,x^*)= [\boldsymbol{h}(x)-\boldsymbol{h}(x^*)] \Delta$. We will consider the case of interactions in Section 3.5.

### 3.2. Illustration

To illustrate the relationship between the EMCs and causal mediation effects, consider the full model with a quadratic effect of the exposure, $\boldsymbol{h}(\boldsymbol{X})=[X,X^2]$, so that the full model is given by $\text{E}[Y|X,M] = \beta_0 + \beta_X X + \beta_{X^2} X^2 + \beta_M M$, the model for $M$ is $\text{E}[M|X] = \alpha_0 + \alpha_X X$, and the submodel for the total effect of $X$ is $\text{E}[Y|X] = \beta_0^* + \beta_X^* X + \beta_{X^2}^* X^2$. The CDE and NDE both equal $\beta_X(x-x^*) + \beta_{X^2}(x-x^*)^2$, which are estimated from the full model. The EMCs $\Delta = \left[ {\matrix{\beta_X^* - \beta_X \cr \beta_{X^2}^* - \beta_{X^2} \cr }}\right] = -\boldsymbol{V_{\boldsymbol{X}M}}\boldsymbol{V_{M}^{-1}}\boldsymbol{\beta_M}$ and the NIE $[\boldsymbol{h}(x)-\boldsymbol{h}(x^*)] \Delta = [x-x^*,x^2-x^{*2}] \left[ {\matrix{\beta_X^* - \beta_X \cr \beta_{X^2}^* - \beta_{X^2} \cr }}\right] = (\beta_X^* - \beta_X)(x-x^*) + (\beta_{X^2}^* - \beta_{X^2})(x^2 - x^{*2})$ are functionals that can be estimated from components of the full model. Notice that the causal mediation effects depend on the choice of $(x,x^*)$, while if $X$ is binary this reduces to $(\beta_X^* + \beta_{X^2}^*) - (\beta_X + \beta_{X^2})$.

Now suppose the full model includes an exposure–mediator interaction so that the full model is $\text{E}[Y|X,M] = \beta_0 + \beta_X X + \beta_{X^2} X^2 + \beta_M M + \beta_{XM} XM$. The implied submodel is $\text{E}[Y|X] = \gamma_0 + \gamma_X X + \gamma_{X^2} X^2$. The EMCs $\Delta = \left[ {\matrix{\gamma_X - \beta_X \cr \gamma_{X^2} - \beta_{X^2} \cr }}\right]$ and the NIE is $[\boldsymbol{h}(x)-\boldsymbol{h}(x^*)]\Delta = (\gamma_X - \beta_X)(x-x^*) + (\gamma_{X^2} - \beta_{X^2})(x^2 - x^{*2})$. For a unit change in $X$, this reduces to $(\gamma_X + \gamma_{X^2}) - (\beta_X + \beta_{X^2})$. Notice that in both examples, the implied submodel $\text{E}[Y|X]$ has the same form. As a result, the total effects $\text{TE}_1 = \beta_X^*(x-x^*) + \beta_{X^2}^*(x^2-x^{*2})$ and $\text{TE}_2 = \gamma_X(x-x^*) + \gamma_{X^2}(x^2-x^{*2})$ would have the same empirical estimate even though the system of equations is different.

### 3.3. The conditional and the unconditional variance of the indirect effect

A closed-form expression for the fully conditional variance of the EMCs follows directly as $\text{Var}(\widehat{\Delta}|\boldsymbol{X},\boldsymbol{M}) = \boldsymbol{V_{XM}} \boldsymbol{V_M^{-1}} \boldsymbol{V_{MX}}$. The variance of the NIE (and more generally, the PE) is trivial to obtain using $[\boldsymbol{h}(x)-\boldsymbol{h}(x^*)]\text{Var}(\widehat{\Delta}|\boldsymbol{X},\boldsymbol{M})[\boldsymbol{h}(x)-\boldsymbol{h}(x^*)]'$, which requires fitting only one model (3.1). From properties of a regression model, for a scalar PE with a unit change in the exposure, we have $\frac{\widehat{\text{PE}} - \text{PE}}{\sqrt{\widehat{\text{Var}}(\widehat{\text{PE}})}} \sim t(\text{df = }n-k,\text{scale}=-1)$ and the 95% CI is $\widehat{\text{PE}} \pm t_{.975,n-k} \times$ -$\hat{V}_{XM} \hat{V}_{M}^{-1} \sqrt{\widehat{\text{Var}}(\hat{\beta}_M)}$.

In equations (3.1) and (3.2), $Y$ is a random variable and $X$ and $M$ are fixed covariates. One may wish to treat the mediator as a random variable and marginalize over $M$. The causal inference framework uses the marginal variance for inference (VanderWeele, 2015). Using the law of total probability,

(3.5) \begin{align*} \text{Var}(\widehat{\Delta}|X) &= \text{E}_{M|X}[\text{Var}(\widehat{\Delta}|X,M)] + \text{Var}_{M|X}[\text{E}(\widehat{\Delta}|X,M)] \nonumber \\ &= \text{E}_{M|X}\left[\frac{n^2 r_{XM}^{2} \hat{\sigma}_M^2 \hat{\sigma}_{Y|X,M}^2}{|\boldsymbol{D}'\boldsymbol{D}|}\right] + \beta_M^2 \left[\frac{\sigma_{M|X}^2}{n \hat{\sigma}_X^2} \right] \label{marginalVar}, \end{align*}

where $\boldsymbol{D} = (1,X,M)$ is the $n\times 3$ design matrix. This quantity can be estimated by plugging in the sample correlation $r$, the maximum likelihood estimates of the variances of $X$ and $M$, estimates of the mean square error of $Y$ from (2.1) and of $M$ from (2.2), and $\hat{\beta}_M$. Notice that the second term in (3.5) is an increasing function of $\beta_M$ and a decreasing function of the sample size $n$. We used simulations to empirically verify (3.5) under various sample sizes $(n = 100, 200, 400, 1000)$ and magnitudes of $\beta_M = 2, 4$. Although the second term in Var($\widehat{\Delta}|X)$ requires estimating the variance of the residuals from the regression of $M$ on $X$, the contribution is of order $1/n$ and becomes negligible in moderate sample sizes. The marginal variance of mediation effects follows.

Because the mediator is (in theory) a consequent of the exposure, $M$ cannot be randomized and one could argue in favor of treating both $X$ and $M$ as fixed (Pearl 2012a). In classical regression settings, the conditional variance is frequently used for inference even when the covariate changes in a population. As we note above, the distinction between the two variances becomes semantic in large samples. Which variance is to be preferred deserves consideration, but further discussion is beyond the scope of this article. Note that the nonparametric bootstrap, which samples with replacement from pairs of $X$ and $M$, approximates the fully unconditional variance (marginalized over both exposure and mediator).

### 3.4. Multiple mediators

**Fig. 3.**
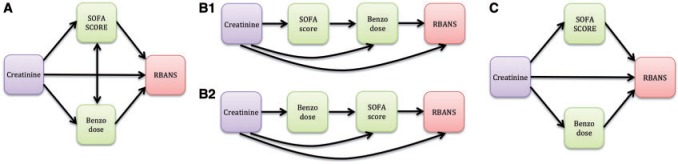
Comparison of multiple mediator models. Model A depicts the proposed single-model framework for assessing mediation with multiple mediators. Model B depicts the serial multiple mediator model. Model C depicts the single-step or parallel multiple mediator model. The directions of arrows indicate the assumed causal pathways.

Suppose the exposure’s effect on the outcome is transmitted through several mediators. Estimating the *total indirect effect* in a multiple mediator model aims to determine whether the *set* of $j$ mediators $\boldsymbol{M}$ transmits the effect of $X$ to $Y$. To identify the NDE and NIE from a multiple mediator model, all four no unmeasured confounding assumptions outlined in Figure 2 must hold with respect to $\boldsymbol{M}$. Existing approaches in the context of multiple mediators include the *single-step multiple mediator model* (MacKinnon, 2008), also termed the *parallel multiple mediator model* (Hayes, 2013), and the *serial multiple mediator model* (Hayes, 2013). The single-step approach specifies a separate outcome model for each mediator in which they independently affect the outcome (see Figure 3C). The serial model relies on assumptions about the directionality of the mediators, which can be unverifiable with cross-sectional data (see Figure 3B). VanderWeele and Vansteelandt (2013) provide both regression-based and weighting approaches that allow mediators to be interdependent (see Figure 3A). The simulation-based approach by Imai *and others* (2010) handles multiple mediator models of all types, but the software currently accommodates only two mediators, and the user must specify one mediator as “main” and the other as “alternative.”

Within our framework, incorporating multiple mediators is simple and efficient. Our formulation allows the mediators to covary, a more realistic assumption than assuming the mediators do not affect each other (as is required for the single-step models) or that we know the order in which they affect each other (as is required for serial models). The advantage of using our approach is that it requires fitting only one model to obtain causal mediation estimands (compared with three or more models required by existing approaches), and it yields model-based variance estimates that do not require the computation time of resampling methods.

If we posit $j$ mediators such that the full mediation model is $\text{E}[Y|X,M] = \beta_0 + \beta_X X + \Sigma_{i=1}^{j}\beta_{M_i} M_i$ and the corresponding submodel for the total effect of $X$ is $\text{E}[Y|X] = \beta_0^* + \beta_X^* X$, then the total indirect effect through $\boldsymbol{M}$ is estimated by $[\boldsymbol{h}(x)-\boldsymbol{h}(x^*)] \hat{\Delta} =$ -$\boldsymbol{\hat{V}}_{X\boldsymbol{M}} \boldsymbol{\hat{V}}_{\boldsymbol{M}}^{-1}\boldsymbol{\hat{\beta}_M} (x-x^*)$ and its variance by $\widehat{\text{Var}}([\boldsymbol{h}(x)-\boldsymbol{h}(x^*)] \hat{\Delta}) = (x-x^*)^2 \boldsymbol{\hat{V}}_{X\boldsymbol{M}} \boldsymbol{\hat{V}}_{\boldsymbol{M}}^{-1}\boldsymbol{\hat{V}}_{\boldsymbol{M}X}$. The *mediator-specific indirect effect* represents the ability of $M_i$ to mediate the effect of $X$ on $Y$ above and beyond the other $j-1$ mediators. The specific indirect effect through $M_{i'}$ is $(\gamma_{X_{i'}} - \beta_X) (x-x^*)$, which is easily estimated using -$\hat{V}_{XM_{i'}} \hat{V}_{M_{i'}}^{-1} \hat{\beta}_{M_{i'}} (x-x^*)$. The specific indirect effect through $M_{i'}$ is estimated using -$\hat{V}_{XM_{i'}} \hat{V}_{M_{i'}}^{-1} \hat{\beta}_{M_{i'}} (x-x^*)$. The variance is estimated with $\hat{V}_{XM_{i'}} \hat{V}_{M_{i'}}^{-1} \hat{V}_{M_{i'} X} (x-x^*)^2$. Importantly, formula 3.3 gives us these effects and their variances without having to fit any submodels.

If two or more mediators share a role in transmitting the effect of $X$ to $Y$, then the effect attributed to a specific mediator $M_i$ may exclude this overlapping effect. Additionally, specific indirect effects might have different signs, leading to inconsistent mediation. As a result, the specific indirect effects attributed to each mediator do not necessarily sum to the total indirect effect mediated by the set of mediators. We emphasize that the estimated total indirect effect through $\boldsymbol{M}$ comes from the full model containing all of the mediators and does not suffer bias from the mis-specification of intermediator relationships. Thus, we recommend the researcher’s primary interest lie in the total indirect effect rather than the amount mediated by a specific mediator. We present relevant examples in Section 6.

The regression-based approach by VanderWeele and Vansteelandt (2013) uses one outcome model for all of the mediators but also requires a separate model for each mediator and each mediator–mediator interaction. Including covariates $C$ can lead to compatibility issues between the models for $M_i$, $M_k$, and their product $M_i M_k$. Their alternative inverse probability weighting approach circumvents this issue in settings with mediator–mediator interactions. The weighting approach allows the mediators to affect each other and does not require modeling the mediators, but it does require fitting several logistic regression models to estimate $\text{P}[X=x]$, $\text{P}[X=x^*]$, $\text{P}[X=x|C=c]$, $\text{and P}[X=x^*|C=c]$ for the weights. It should be noted that the weighting method performs best when the exposure has only a few levels (e.g., binary or discrete) (VanderWeele and Vansteelandt, 2013).

### 3.5. Interactions and moderated mediation

Our framework accommodates exposure–exposure and mediator–mediator interactions as well as interactions with confounders. Simply include the interaction terms of interest in the full model and use formulas 3.3 and 3.4 to estimate the EMCs and causal mediation effects. We now consider the more complex setting of exposure–mediator interactions (so-called *moderated mediation*).

The causal mediation literature often considers exposure–mediator interactions with binary $X$, such that the full model is $\text{E}[Y|X,M] = \beta_0 + \beta_X X + \beta_M M + \beta_{XM} XM$ and the submodel is $\text{E}[Y|X] = \beta_0^* + \beta_X^* X$. The portion eliminated PE = TE$-$CDE($m)= [\beta_X^* - (\beta_X + \beta_{XM}m)](x-x^*)$ is estimated from the fit of the full model using $[\Delta - \beta_{XM}m](x-x^*)$. One could plot the PE as a function of $M$ or report the PE for a point of interest $m$, such as the sample mean. Its variance follows by direct calculation: $(x-x^*)^2 \left[V_{XM} V_{M}^{-1} V_{MX} + m^2 \text{Var}(\hat{\beta}_{XM}) - 2m V_{XM} V_{M}^{-1} \text{Cov}(\hat{\beta}_M, \hat{\beta}_{XM})\right]$.

If the exposure is continuous, then the exposure–mediator interaction model above implies a marginal model that includes an $X^2$ term. In this case, the marginal model cannot be estimated using the single-model framework, because it is not nested within the full model. Our framework requires that the full model also include an $X^2$ term (see Section 3.2 for an example), which can be viewed as relaxing the assumption that all nonlinear effects of the exposure act through the mediator. In this sense, a broader full model is desirable. Despite the non-nested submodel, it is possible to use the mediation formula to proceed with estimation in this setting. Further thoughts are included in Remark D.

Notice that exposure–mediator interactions lead to mediation effects that are less clearly defined. Because $M$ acts simultaneously as a moderator and a mediator, both the direct and indirect effects are affected by $\beta_{XM}$. As a result, there is more than one way to decompose the total effect depending on how the interaction effect is accounted for (Robins and Greenland, 1992; Pearl, 2001). If one attributes $\beta_{XM}$ to the indirect effect, then the total effect decomposes into the NDE and total indirect effect. Conversely, if one attributes $\beta_{XM}$ to the direct effect, then the total effect decomposes into the total direct effect and pure indirect effect. Thus, mediation and moderation are “inextricably intertwined and cannot be assessed separately” (Pearl, 2012b).

With exposure–mediator interactions, the CDE and NDE diverge: CDE($m$) = $(\beta_X + \beta_{XM}m)(x-x^*)$ and NDE = $(\beta_X + \beta_{XM}\text{E}[M|x^*])(x-x^*)$. Because the CDE is a function of $m$, the PE and its variance are functions of the mediator as well. The NDE marginalizes over $\text{E}[M|x^*]$ and is a function of the exposure that can be defined for any levels of $(x,x^*)$ (Naimi *and others*, 2014). The PE does not depend on the choice of decomposition, because it is the portion of the total effect attributed to *both* interaction and mediation. As such, the PE is a comprehensive estimate of moderated mediation in these complex settings.

## 4. Impact of omitted covariates on estimating the indirect effect

What happens to $\widehat{\Delta}$ when we omit an important covariate in the specification of the full model? If the omitted covariate is orthogonal to $X$ or to $M$, then $\widehat{\Delta}$ does not incur additional bias. To fix ideas, consider the simple setting in which we have one exposure $X$, one mediator $M$, and a third omitted covariate $W$. Suppose the true data-generating mechanism is $Y = \gamma_0 + \gamma_X X + \gamma_M M + \gamma_W W + \varepsilon$, but we do not know $W$ so we *incorrectly* specify the full model as $Y = \beta_0 + \beta_X X + \beta_M M + \varepsilon$ and the submodel as $Y = \beta_0^* + \beta_X^* X + \varepsilon$. The estimates $\hat{\beta}_X$ and $\hat{\beta}_X^*$ will be biased estimates of $\gamma_X$, the “true” effect of $X$. The expected values of the parameters from the full model and submodel are $\left[ {\matrix{\hat{\beta}_0 \cr \hat{\beta}_X \cr \hat{\beta}_M \cr }}\right] \rightarrow E_{true} \left[ {\matrix{\hat{\beta}_0 \cr \hat{\beta}_X \cr \hat{\beta}_M \cr }}\right] = \left[ {\matrix{\gamma_0 \cr \gamma_X \cr \gamma_M \cr }}\right] + \left[ {\matrix{\delta_0 \cr r_{WX.M} \sigma_W / \sigma_X \cr r_{WM.X} \sigma_W / \sigma_M \cr }}\right] \gamma_W$ and $\left[ {\matrix{\hat{\beta}_0^* \cr \hat{\beta}_X^* \cr }}\right] \rightarrow E_{true} \left[ {\matrix{\hat{\beta}_0^* \cr \hat{\beta}_X^* \cr }}\right] = \left[ {\matrix{\gamma_0 \cr \gamma_X \cr }}\right] + \left[ {\matrix{\alpha_0 \cr r_{XM} \sigma_M / \sigma_X \cr }}\right] \gamma_M + \left[ {\matrix{\kappa_0 \cr r_{XW} \sigma_W / \sigma_X \cr }}\right] \gamma_W$, respectively. Thus, when we omit $W$, the expected difference in the estimated total and direct effects for a unit change in $X$ is $\text{E}[\widehat{\Delta}_1] = E_{\text{true}}[\hat{\beta}_X^* - \hat{\beta}_X] = r_{XM}\frac{\sigma_M}{\sigma_X} \gamma_M + (r_{XW} - r_{XW.M})\frac{\sigma_W}{\sigma_X} \gamma_W$.

Next, suppose that $Y$ does not depend on $W$ and the true data-generating mechanism is $Y = \gamma_0 + \gamma_X X + \gamma_M M + \varepsilon$. If we *correctly* specify the full model as $Y = \beta_0 + \beta_X X + \beta_M M + \varepsilon$ and the submodel as $Y = \beta_0^* + \beta_X^*X + \varepsilon$, then $\text{E}[\widehat{\Delta}_2] = \text{E}[\hat{\beta}_X^* - \hat{\beta}_X] = \gamma_X + (X'X)^{-1}X'M \gamma_M - \gamma_X = r_{XM}\frac{\sigma_M}{\sigma_X} \gamma_M$. The bias in the estimated indirect effect when the full model omits $W$ is given by $\text{E}[\widehat{\Delta}_1 - \widehat{\Delta}_2] = (r_{XW} - r_{XW.M})\frac{\sigma_W}{\sigma_X} \gamma_W$. As a result, if $W$ is orthogonal to $X$ or $M$ ($r_{XW} = r_{XW.M}$) or $\gamma_W=0$ (a trivial case), then the estimated indirect effect under the incorrectly specified full model is robust to mis-specification. That is, omitting $W$ will not change the estimate of the indirect effect.

Note that if $W$ is *not* orthogonal to either $X$ or $M$ such that $W$ is a confounder of the exposure–mediator relationship, then assumption (iii) of the no-unmeasured confounding assumptions is violated and so the NDE and NIE are not identifiable. The CDE is still identifiable in this setting, provided there are no unmeasured confounders of the exposure–outcome and mediator–outcome relationship (see Figure 2).

## 5. Simulations

### 5.1. Setup

We use simulations to provide empirical support for the proposed approach to mediation analysis with a simple mediation model. We simulated 5000 data sets of sample size $n \in \{50,100,200\}$ with a “true” indirect effect of 1.5. The “true” full model was $Y = \beta_0 + \beta_X X + \beta_M M + \varepsilon_Y$, where $\varepsilon \sim N(0,\sigma_Y^2)$. The exposure $X \sim N(0, \sigma_X^2)$ and mediator $M = \alpha_0 + \alpha_X X + \varepsilon_M$, where $\varepsilon_M \sim N(0,\sigma_M^2)$. When comparing methods, 10 000 bootstrap replications and 10 000 Monte Carlo draws were used. For each replication, we computed each method’s estimated indirect effect and estimated variance and compared these with the true effect and the empirical (“true”) variance. The bias of the estimated indirect effect was captured when $X$ and $M$ were both fixed and when $M$ was random. These simulations demonstrate the performance of our formulas and are not intended to be exhaustive. Varying parameter values affected the magnitude of the results but not the general patterns.

### 5.2. How our variance measure compares with the existing measures

The results of estimating the variance of the indirect effect using the analytical regression-based formula, bootstrapping, Sobel’s formula, and Monte Carlo methods are shown in Figure 1 and Table 1 of supplementary material available at *Biostatistics* online. The analytical variance formula appears unbiased for the true variance. Sobel’s variance performs similarly to the case bootstrap. As expected, the estimated variance from bootstrapping cases is greater than that from the residual bootstrap (see Section 3.3). As the sample size increases, the variances of the estimates of $\widehat{\text{Var}}([h(x) - h(x^*)]\widehat{\Delta})$ from the cases bootstrap, Sobel’s formula, and the MC methods decrease but remain biased.

### 5.3. The bias of indirect effect estimates depends on the conditioning set

Under the full model, the expectation of $\widehat{\Delta}$ is $\text{E}[\hat{\beta}_X^* - \hat{\beta}_X] = [\beta_X + (X'X)^{-1}X'M\beta_M] - \beta_X = P_{X.M} \beta_M$, where $P_{X.M}$ is the projection of $M$ onto $X$. For the simple mediation model, $P_{X.M} \beta_M = \rho_{XM} \left(\frac{\rho_{MY} - \rho_{YX} \rho_{XM}}{1-\rho_{XM}^2} \frac{\sigma_Y}{\sigma_X} \right)$. To estimate $\widehat{\Delta}$, we replace $\rho$ and $\sigma^2$ with their sample estimates $r$ and $s^2$ to obtain $r_{XM} \left(\frac{r_{MY} - r_{YX} r_{XM}}{1-r_{XM}^2} \frac{s_Y}{s_X} \right)$, which is biased per Jensen’s inequality. This is not surprising, because the sample correlation $r$ is a biased estimate of $\rho$, a result given by Fisher (1915): $\text{E}[r] = \rho - \rho(1-\rho^2)/2N$. Since $r \rightarrow \rho$ and $s^2 \rightarrow \sigma^2$ as $N \rightarrow \infty$, $\widehat{\Delta}$ is biased but consistent for the true $\Delta$ by the Law of Large Numbers and the Continuous Mapping Theorem.

The distributions of $\widehat{\Delta}$ when $X$ and $M$ are both fixed and when $M$ varies are shown in Figure 2 of supplementary material available at *Biostatistics* online (note that $\widehat{\Delta}$ equals the indirect effect for a unit change in $X$ from the simple mediation model). When $X$ and $M$ are both fixed, $\widehat{\Delta}$ is biased as a function of the bias of $r_{XM}$. If we allow $M$ to vary, the bias is reduced because $r_{XM}$ is no longer fixed and it tends to approximate $\rho_{XM}$ better on average. Therefore, because of the bias–variance trade-off, coverage probability alone is not the proper performance measure when $r_{XM}$ poorly approximates $\rho_{XM}$.

## 6. Examples with data from Vanderbilt ICU patients

We illustrate our method and existing approaches using data from a prospective cohort of 217 intensive care unit (ICU) patients at Vanderbilt University Medical Center with acute respiratory failure and/or cardiogenic or septic shock (Pandharipande *and others*, 2013). The goal is to examine the cognitive effects of critical illness. We use measurements of creatinine (mg/dL) and estimated glomerular filtration rate measured at baseline, benzodiazepine dose (mg), Sequential Organ Failure Assessment (SOFA) score, mental status (delirious or normal) assessed with the Confusion Assessment Method for the ICU and Richmond Agitation-Sedation Scale, and Repeatable Battery for the Assessment of Neuropsychological Status (RBANS), a global cognitive score measured three months post-discharge. Biomarker S100B levels were measured for 121 of these patients.

We present several simple examples of mediation models to illustrate the efficiency and coherence of our proposed framework. We compare variance estimates obtained from the model-based formula, Sobel’s formula, and percentiles of 10 000 bootstrap replications. The first two examples assume there are no unmeasured confounders, and the third assumes the covariate $C$ sufficiently controls for confounding. We do not intend for the examples and their results to be interpreted scientifically; rather, they are meant to illustrate the methods discussed throughout the article. All models assume errors $\varepsilon \sim \text{N}(0,\sigma^2)$. Unless otherwise specified, we compare unit changes in the exposure so that $(x-x^*) = 1$.

Example 1 (Simple mediation model) Does severity of illness (SOFA) mediate the effect of creatinine on S100B levels? We specify the full model as $\texttt{S100B} = \beta_0 + \beta_X \texttt{Cr} + \beta_M \texttt{SOFA} + \varepsilon$ to estimate the EMC $\widehat{\Delta}$ = -$\hat{V}_{XM} \hat{V}_{M}^{-1} \hat{\beta}_M$, where $X = \texttt{Cr}$ and $M=\texttt{SOFA}$. The mediated effect of creatinine on S100B is $\widehat{\Delta}(x-x^*)=28.64$ (SE = 7.12) with 95% 14.54, 42.74. The residual bootstrap SE = 7.06, Sobel’s SE = 17.25, and the case bootstrap SE = 18.74. Importantly, the model-based variance is five times smaller than Sobel’s and the case bootstrap, which yield 95% CIs that include zero. Although the residual bootstrap variance gives essentially the same answer as the model-based formula, the formula avoids the computation time and effort.

To allow for a *quadratic* relationship between creatinine and S100B, simply specify the full model as $\texttt{S100B} = \beta_0 + \beta_{X_1} \texttt{Cr} + \beta_{X_2} \texttt{Cr}^2 + \beta_M \texttt{SOFA} + \varepsilon$. The EMCs $\widehat{\Delta}$= -$\hat{V}_{\boldsymbol{X}M} \hat{V}_{M}^{-1} \hat{\beta}_M = \left[ {\matrix{28.33 \cr -11.08 \cr }}\right]$ is now a vector of the linear and quadratic effects of creatinine. The PE (which equals the NIE) is 28.33$(x-x^*) - 11.08(x^2 - x^{*2}) = 17.25$. Using the mediation package gives an estimated NIE of 17.25 (exactly equal to our estimate, as expected) and requires 111.94 s of computation time compared to 0.01 s using our approach. In the remaining examples, we consider only linear effects but allowing for nonlinear relationships in practice is strongly advised and easily implemented within the proposed framework.

Example 2 (Simple mediation model where Sobel’s approximation holds) It is not always true that we see such large efficiency gains. For instance, our method yields similar results to standard approaches when we investigate whether the relationship between creatinine and overall cognitive function (RBANS) is mediated by severity of illness. The estimated indirect effect is 0.01 (SE = 0.27), the residual bootstrap SE = 0.27, Sobel’s SE = 0.27, and the case bootstrap SE = 0.29.

Example 3 (Exposure-confounder interactions) Recall that identifying mediation effects relies on a strict set of no unmeasured confounding assumptions (outlined in Figure 2). To keep this example simple, we assume that adjusting for $C$ = Charlson score is sufficient to satisfy these assumptions. We also include an exposure–confounder interaction, so that the full model is $\texttt{RBANS} = \beta_0 + \beta_X \texttt{Cr} + \beta_{M} \texttt{SOFA} + \beta_{C} \texttt{Charlson} + \beta_{XC}\texttt{Cr:Charlson} + \varepsilon$. The indirect effect marginalized over the confounder is E$[h(x)-h(x^*)]\Delta|C] = (\beta_{X}^* - \beta_X)(x-x^*) + (\beta_{XC}^* - \beta_{XC})(x-x^*) \text{E}[C]$. The variance is estimated using $(x-x^*)^2 \text{Var}(\Delta_1) + (x-x^*)^2 \text{E}[C]^2 \text{Var}(\Delta_2) + 2(x-x^*)^2\text{E}[C]\text{Cov}(\Delta_1, \Delta_2)$.

For a unit change in creatinine, the estimated indirect effect is 0.0028 (SE = 0.22). The regression-based approach by VanderWeele requires fitting the mediator model $\texttt{SOFA} = \alpha_0 + \alpha_X \texttt{Cr} + \alpha_{C} \texttt{Charlson} + \beta_{XC}\texttt{Cr:Charlson} + \varepsilon$ in addition to the full model. The mediation formula estimates the indirect effect using $\text{E}[\beta_M (\text{E}[M|x] - \text{E}[M|x^*])|C] = \beta_M (\alpha_X + \alpha_{XC}\text{E}[C])(x-x^*) = 0.0028$ (SE = 0.24).

To examine the indirect effect comparing the 75th percentile to the median value of creatinine, one simply plugs in these values for $x$ and $x^*$. Using the single-model approach took 0.02 s to estimate the total, direct, and indirect effects, and an additional 0.003 s to recalculate the indirect effect for the new pair of exposure values. Using the simulation-based mediation package required 24.89 s, and a new simulation must be run for each additional pair of exposure values. Although this difference may seem inconsequential for this simple example, using 3.3 and 3.4 reduces the computation time by several orders of magnitude when applied to big data. For example, with the current sample size (*N* = 217), if one were to study mediation across 10 000 SNPs and three different pairs of the exposure were of interest, the simulation-based approach would require around $10\,000 \times 24.89 \times 3$ s (over 8 days) to run. The proposed approach would take $(10\,000 \times 0.02) + (0.003 \times 3)$ s (under 4 min).

Example 4 (Multiple mediator model) Is the effect of creatinine on cognitive function mediated by severity of illness and benzodiazepine dose? The conceptual diagrams in Figure 3 depict the single-model approach for multiple mediators, the serial multiple mediator model, and the parallel multiple mediator model. For all three methods, the full model is $\texttt{RBANS} = \beta_0 + \beta_X \texttt{Cr} + \beta_{M_1} \texttt{SOFA} + \beta_{M_2} \texttt{Benz} + \varepsilon$ and the submodel for the total effect of creatinine on cognitive function is $\texttt{RBANS} = \beta_0^* + \beta_X^* \texttt{Cr} + \varepsilon$. Thus, the direct effect of creatinine is $\beta_X(x-x^*)$, the total effect of creatinine is $\beta_X^*(x-x^*)$, and the total indirect effect of creatinine through SOFA and benzodiazepine is $(\beta_X^* - \beta_X)(x-x^*)$, regardless of which method you use. It is important to recognize that the total indirect effect does not depend on the order or directionality of the mediators, whereas the amount of mediation attributed *specifically* to SOFA or benzodiazepine will differ across methods due to their varying assumptions about intermediator relationships.

We estimate how much severity of illness and benzodiazepine dose mediate the relationship between creatinine and cognitive function using only the full model and formula 3.4. For $X = \texttt{Cr}$ and $\boldsymbol{M} = \{\texttt{SOFA},\texttt{Benz}\}$, the total indirect effect through $\boldsymbol{M}$ is estimated using $[h(x)-h(x^*)]\widehat{\Delta}$ = -$\hat{V}_{X\boldsymbol{M}} \hat{V}_{\boldsymbol{M}}^{-1}\hat{\beta}_{\boldsymbol{M}}(x-x^*)$ = $-$0.34 and its empirical variance $\hat{V}_{X\boldsymbol{M}} \hat{V}_{\boldsymbol{M}}^{-1} \hat{V}_{\boldsymbol{M}X}(x-x^*)^2$ = 0.139 (SE = 0.37).

Now suppose we are interested in mediator-specific effects. Since we have already fit the full model, to estimate how much is mediated specifically through $M_1 = \texttt{SOFA}$ we simply apply 3.3: -$\hat{V}_{XM_1} \hat{V}_{M_1}^{-1} \hat{\beta}_{M_1}(x-x^*)$ = $-$0.046. The variance follows directly: $\hat{V}_{XM_1} \hat{V}_{M_1}^{-1} \hat{V}_{M_1 X}(x-x^*)^2$ = 0.092 (SE = 0.30). Similarly, to estimate how much the effect of creatinine is mediated through $M_2$ = $\texttt{Benz}$, use -$\hat{V}_{XM_2} \hat{V}_{M_2}^{-1}\hat{\beta}_{M_2}(x-x^*)$ = $-$0.352, which has an estimated variance of $\hat{V}_{XM_2} \hat{V}_{M_2}^{-1} \hat{V}_{M_2X}(x-x^*)^2 =0.066$ (SE = 0.26). Keeping in mind SOFA is correlated with benzodiazepine dose, notice that the mediator-specific indirect effects sum to $-$0.398, which does not equal the total indirect effect of $-$0.34.

The parallel approach (MacKinnon, 2008; Hayes, 2013) and the causal regression-based approach (VanderWeele and Vansteelandt, 2013) specify the same full model as above $\texttt{RBANS} = \beta_0 + \beta_X \texttt{Cr} + \beta_{M_1} \texttt{SOFA} + \beta_{M_2} \texttt{Benz} + \varepsilon$ and an additional model for each mediator: $\texttt{SOFA} = \alpha_{01} + \alpha_{X1} \texttt{Cr} + \varepsilon$ and $\texttt{Benz} = \alpha_{02} + \alpha_{X2} \texttt{Cr} + \varepsilon$. This specification assumes the mediators “act in parallel” (see Figure 3C). The estimated indirect effect through SOFA is $\hat{\alpha}_{X1} \hat{\beta}_{M_1}$ = $-$0.042 (SE = 0.27) and the estimated indirect effect through benzodiazepine is $\hat{\alpha}_{X2} \hat{\beta}_{M_2}$ = $-$0.299 (SE = 0.25), which sum to the total indirect effect. Delta method approximations are used to estimate standard errors. When the exposure is continuous and there are no mediator–mediator interactions, the weighting approach is not recommended (VanderWeele and Vansteelandt, 2013).

The serial model given by Hayes (2013) requires specifying the order in which the mediators affect each other. Suppose we assume $\texttt{Cr} \rightarrow \texttt{SOFA} \rightarrow \texttt{Benz} \rightarrow \texttt{RBANS}$ (see Figure 3B1). The full model is specified as $\texttt{RBANS} = \beta_0 + \beta_X \texttt{Cr} + \beta_1 \texttt{SOFA} + \beta_2 \texttt{Benz} + \varepsilon$ (the same as above), the first submodel is $\texttt{Benz} = \alpha_{02} + \alpha_2 \texttt{Cr} + \delta_{21} \texttt{SOFA} + \varepsilon$, and the second submodel is $\texttt{SOFA} = \alpha_{01} + \alpha_1 \texttt{Cr} + \varepsilon$. There are three estimated indirect effects: $\hat{\alpha}_1 \hat{\beta}_1$ = $-$0.04 (SE = 0.27) is the indirect effect of creatinine through SOFA to RBANS, $\hat{\alpha}_2 \hat{\beta}_2$ = $-$0.35 (SE = 0.29) is the indirect effect of creatinine through benzodiazepine to RBANS, and $\hat{\alpha}_1 \hat{\delta}_{21} \hat{\beta}_2$ = 0.05 (SE = 0.36) is the indirect effect of creatinine through SOFA to benzodiazepine to RBANS. The variances of $\hat{\alpha}_1 \hat{\beta}_1$ and $\hat{\alpha}_2 \hat{\beta}_2$ are estimated using Sobel’s formula and $\widehat{\text{Var}}(\hat{\alpha}_1 \hat{\delta}_{21} \hat{\beta}_2) = \hat{\alpha}_1^2 \hat{\delta}_{21}^2 s^2_{\beta_2} + \hat{\alpha}_1^2 \hat{\beta}_2^2 s^2_{\delta_{21}} + \hat{\delta}_{21}^2 \hat{\beta}_2^2 s_{\alpha_1}^2$ (Hayes, 2013).

To demonstrate how mediator-specific indirect effects depend on the specified order in a serial model, suppose we change the order of mediation to $\texttt{Cr} \rightarrow \texttt{Benz} \rightarrow \texttt{SOFA} \rightarrow \texttt{RBANS}$ (see Figure 3B2). The total indirect effect remains unchanged, but now the indirect effect of creatinine through benzodiazepine to RBANS is $-$0.299, the indirect effect of creatinine through SOFA to RBANS is $-$0.046, and the indirect effect of creatinine through benzodiazepine to SOFA to RBANS is 0.0046. Notice that in either case, the serially mediated indirect effects sum to the total indirect effect. Estimating the indirect effects from the serial model is analogous to examining sequential sums of squares, whereas estimating effects from the proposed framework is analogous to examining partial sums of squares. Just as partial sums of squares do not necessarily sum to the total, mediator-specific indirect effects do not necessarily sum to the total indirect effect. In contrast, the serial indirect effects do sum to the total indirect effect, but their estimation depends heavily on the assumed order of the mediators.

## 7. Remarks

The statistical literature abounds with methods for estimating the indirect effect and its variance from the simple mediation model. For sophisticated mediation analyses involving interactions, splines, and any combination of continuous, binary, and categorical mediators, the proposed single-model approach is straightforward to implement.

*Remark A: Straightforward application of modeling tools*

The proposed framework can be viewed as having two key steps: first, estimation of a single fully conditional model for the outcome and second, estimation of mediation functionals from that model. As a result, this approach allows for mediation analysis with a straightforward application of regression modeling tools—e.g., penalization procedures such as the elastic net or lasso, multiple imputation, and cross-validation. One simply applies these techniques to the single well-specified full model and their impact is automatically incorporated in the mediation functionals.

*Remark B: Advantage of using one outcome model in multiple mediator settings*

As pointed out by VanderWeele and Vansteelandt (2013), the approach of using one outcome model for all of the mediators is “robust to unmeasured common causes $[C]$ of two or more mediators,” whereas having separate outcome models for each mediator is not. When the outcome model contains all the mediators, $C$ only affects the outcome through the set of mediators, so $C$ does not confound the joint effect of $\boldsymbol{M}$ on $Y$. If, instead, one specifies a separate outcome model for each mediator, $C$ affects $M_i$ and it affects $Y$ through $\boldsymbol{M}_{i'\neq i}$, which leads to biased estimates of the the effect $M_i$ on $Y$. Thus, it is recommended to specify one full outcome model that contains all of the mediators.

*Remark C: Controlled indirect effect*

“Controlled indirect effects are notably difficult to conceptualize, and instead are defined as some contrast between the total and controlled direct effects in the absence of exposure-mediator interactions” (Naimi *and others*, 2014). Our approach provides a general formula for estimating the difference between the total effect and the CDE, i.e., the so-called controlled indirect effect. By contrast, the mediation formula provides a general formula for estimating the NIE, the difference between the total effect and the NDE (Pearl, 2001).

*Remark D: Non-nested submodels*

To use the single-model approach, the implied submodel must be nested within the full model. The simple exposure–mediator interaction model is a commonly encountered example of a marginal model that is not nested. The full model $\text{E}[Y|X,M] = \beta_0 + \beta_X X + \beta_M M + \beta_{XM}XM$ has the implied submodel $\text{E}[Y|X] = \beta_0^* + \beta_X^* X + \beta_{X^2}^* X^2$. This full model contains a linear term for $X$, which implies the entire nonlinear effect of the exposure is captured by the mediator (via the interaction). This is an impactful assumption that we would prefer to relax by including the nonlinear exposure effects $h(X)$ in both the full and marginal models.

*Remark E: Fitted versus implied total effect*

The standard approach in the causal inference literature is to use the implied total effect that results from fitting the full outcome model and the model for the mediator. We say “implied” here, because the marginal model $\text{E}[Y|X]$ is never actually fit to the data. Instead, the sum of the estimated NDE and NIE is used as the total effect estimate (e.g., this is how the mediation package in R estimates the total effect). In contrast, our approach estimates the total effect directly from the fitted marginal model.

Importantly, the estimated total effect obtained from *fitting* the marginal model $\text{E}[Y|X]$ does not necessarily equal the sum of the estimated NDE and NIE, an unexpected finding. We found this to be the case when fitting the full model $\text{E}[Y|X,M] = \beta_0 + \beta_X X + \beta_M M + \beta_{XM} XM$, the mediator model $\text{E}[M|X] = \alpha_0 + \alpha_X X$, and the implied marginal model $\text{E}[Y|X] = \gamma_0 + \gamma_X X + \gamma_X^2 X^2$. To be clear, our empirical estimate of the total effect $\gamma_X(x-x^*) + \gamma_X^2(x^2 - x^{*2})$ did not equal the sum of the NDE and NIE. We can only speculate that the maximum likelihood fit of the submodel is not equivalent to the implied submodel derived from the maximum likelihood fits of the first two models. One explanation is that several different systems of equations will yield the same submodel, but only one submodel is implied once the outcome model and mediator model are fit. This is an interesting finding that merits further study.

## 8. Software

While the BRAIN-ICU data used for the examples are not publicly available, software in the form of R code and documentation is available at https://github.com/trippcm/Biostatistics-Mediation-R-Code

## Supplementary Material

Supplementary Data
