# Supplementary material for: A classical regression framework for mediation analysis: fitting one model to estimate mediation effects
Source: Biostatistics. 2017 Oct 26;19(4):514–28. doi: 10.1093/biostatistics/kxx054 (PMC6180946; doi:10.1093/biostatistics/kxx054)
Supplement: Supplementary Data [file kxx054_tripp_biostatistics_supplement_v2.pdf]

Biostatistics (2017), **0**, 0, *pp.* 1–8
doi:10.1093/biostatistics/Tripp˙biostatistics˙supplement˙v2

Supplementary Material to A Classical Regression Framework for Mediation Analysis: Fitting One Model to Estimate Mediation Effects

CHRISTINA T. SAUNDERS*, JEFFREY D. BLUME*

Department of Biostatistics, Vanderbilt University, Nashville, Tennessee, U.S.A.

christina.m.tripp@vanderbilt.edu, j.blume@vanderbilt.edu

Summary

Mediation analysis explores the degree to which an exposure's effect on an outcome is diverted through a mediating variable. We describe a classical regression framework for conducting mediation analyses in which estimates of causal mediation effects and their variance are obtained from the fit of a single regression model. The vector of changes in exposure pathway coefficients, which we named the Essential Mediation Components (EMCs), is used to estimate standard causal mediation effects. Because these effects are often simple functions of the EMCs, an analytical expression for their model-based variance follows directly. Given this formula, it is instructive to revisit the performance of routinely used variance approximations (e.g., delta method and resampling methods). Requiring the fit of only one model reduces the computation time required for complex mediation analyses and permits the use of a rich suite of regression tools that are not easily implemented on a system of three equations, as would be required in the Baron-Kenny framework. Using data from the BRAIN-ICU study, we provide examples to illustrate the advan-

*To whom correspondence should be addressed.

© The Author 2017. Published by Oxford University Press. All rights reserved. For permissions, please e-mail: journals.permissions@oup.com

tages of this framework and compare it to existing approaches.

Key words: Direct and indirect effect; Intermediate variable; Mediation analysis; Multiple mediators; Regression.

1. Web Appendix A

Our general form for the difference of coefficients approach provides estimates of the portion eliminated, which is the difference between the total effect and the controlled direct effect. Here, we provide a few relevant quotes that highlight the utility of the portion eliminated from a health policy perspective.

"If the policy evaluated aims to prevent the outcome Y by way of weakening the mediating pathways, the target of analysis should be the difference TE − DE, which measures the highest prevention potential of any such policy. This maximum will be realized when the mediating path is totally suppressed, thus reducing the total effect from TE to DE, hence the difference TE − DE" (Pearl 2012).

"The use of natural direct and indirect effects is sometimes criticized because (1) they require very strong assumptions for identification and (2) they do not correspond to any particular intervention that we could actually carry out (this is because they require, for each person, fixing the mediator to the counterfactual level the person would have had in the absence of exposure). Such effect estimates do not correspond to actual interventions we could carry out in practice, and are thus of limited interest from a policy perspective" (VanderWeele 2013).

"We further note that public health questions may be better served by estimating controlled direct effects... As practitioners of the science of public health, it has long been argued that epidemiologists should seek to estimate parameters that have a logical correspondence with some realistic intervention that might be taken to improve population health... Some have argued that

because natural direct and indirect effects cannot be identified using intervention-based causal models, and cannot be estimated in a randomized trial, they cannot be interpreted as effects that have a logical correspondence with some public health action or policy.... Rather than estimating natural effects, epidemiologists may be better off estimating controlled direct effects. These effects correspond to the exposure effect that would remain after an intervention that sets the mediator to a specific level. Such effects are well suited to epidemiology conducted in the context of public health research, in that they seek to understand the change in the outcome that might be brought about by intervening on the exposure and mediator under study" (Naimi et al 2014).

The portion eliminated "is attractive because it concerns the effect of actual potential policy interventions and because it requires only the estimation of controlled direct effects, which, as we have seen, can be identified under somewhat weaker assumptions than natural direct and indirect effects" (VanderWeele 2015).

2. Web Appendix B

Pearl's mediation formula is given by

$$\text{NDE}(x, x^*) = \Sigma_{c,m} \left(\text{E}[Y|x, m, c] - \text{E}[Y|x^*, m, c] \right) \text{P}[m|x^*, c]\text{P}[c]$$

$$\text{NIE}(x, x^*) = \Sigma_{c,m} \text{E}[Y|x, m, c] \left(\text{P}[m|x, c] - \text{P}[m|x^*, c] \right) \text{P}[c]$$

$$\text{TE}(x, x^*) = \Sigma_{c} \left(\text{E}[Y|x, c] - \text{E}[Y|x^*, c] \right) \text{P}[c]$$

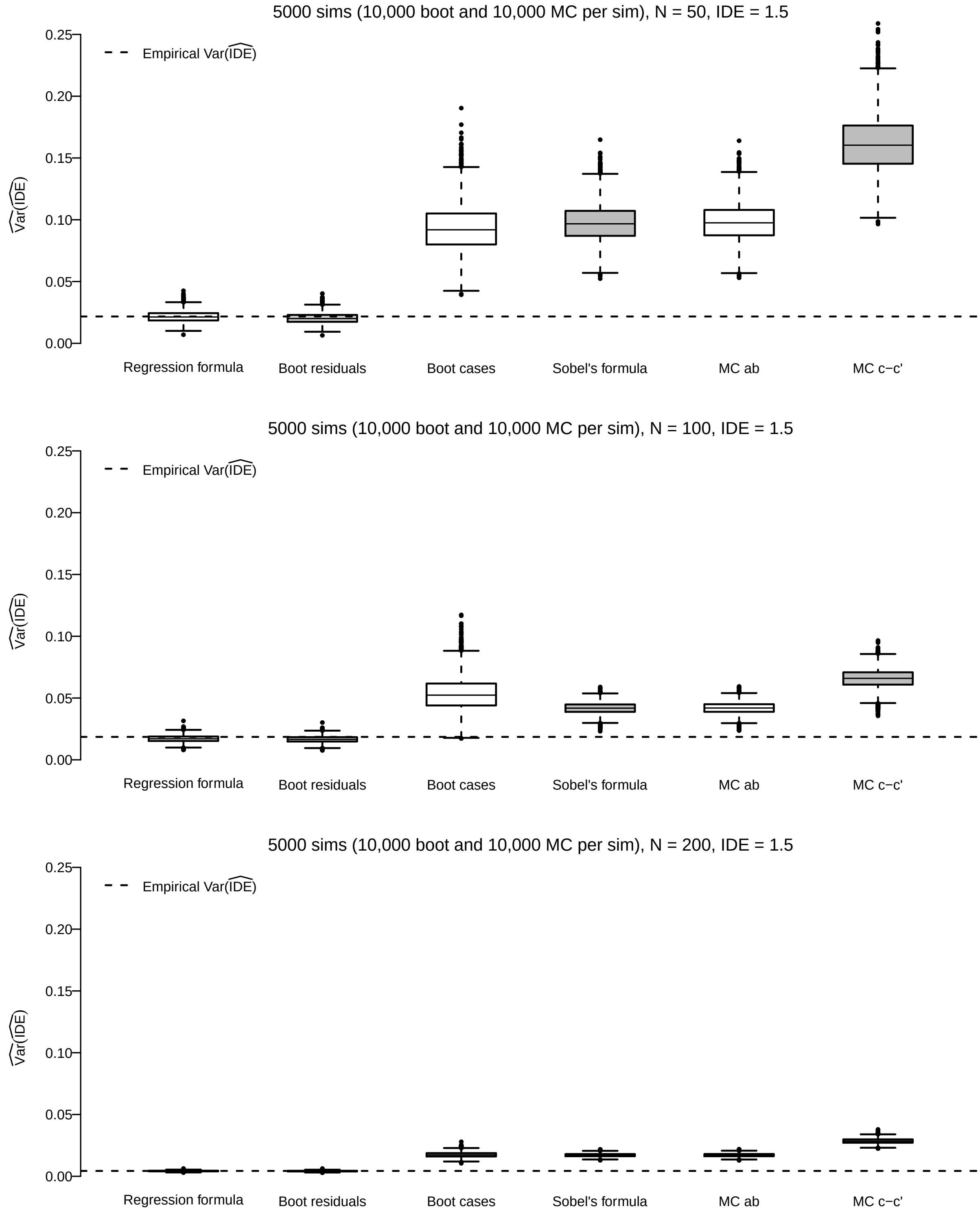

Fig. 1. Results of simulating the estimated variance of indirect effect estimates from the simple mediation model for sample sizes $N = 50, 100, 200$. Note that for the simple mediation model, the (natural) indirect effect for a unit change in the exposure equals the Essential Mediation Component Δ. We use $\widehat{\text{IDE}}$ as an acronym for the indirect effect. We compare $\widehat{\text{Var}}(\widehat{\text{IDE}})$ using the analytical regression formula, bootstrapping residuals and cases, Sobel's formula, and Monte Carlo methods for the product and difference of coefficients.

3. Web Table 1

4. Web Table 2

5. Web Figure 1

6. Web Figure 2

[*Received August 1, 2010; revised October 1, 2010; accepted for publication November 1, 2010*]

N=50	Avg $\widehat{\text{IDE}}$	Bias $\widehat{\text{IDE}}$	Var $\widehat{\text{IDE}}$	Avg $\widehat{\text{Var}}(\widehat{\text{IDE}})$	Bias $\widehat{\text{Var}}(\widehat{\text{IDE}})$
Regression formula	1.5541	0.0541	0.0217	0.0217	-0.0001
Sobel's formula	1.5541	0.0541	0.0217	0.0975	0.0758
Bootstrap cases	1.5375	0.0375	0.0213	0.0931	0.0714
Bootstrap residuals	1.5541	0.0541	0.0217	0.0203	-0.0014
MC difference of coef	1.5540	0.0540	0.0217	0.1616	0.1398
MC product of coef	1.5540	0.0540	0.0217	0.0982	0.0765

N=100	Avg $\widehat{\text{IDE}}$	Bias $\widehat{\text{IDE}}$	Var $\widehat{\text{IDE}}$	Avg $\widehat{\text{Var}}(\widehat{\text{IDE}})$	Bias $\widehat{\text{Var}}(\widehat{\text{IDE}})$
Regression formula	1.8430	0.3430	0.0185	0.0171	-0.0015
Sobel's formula	1.8430	0.3430	0.0185	0.0417	0.0231
Bootstrap cases	1.8462	0.3462	0.0188	0.0533	0.0347
Bootstrap residuals	1.8430	0.3430	0.0185	0.0165	-0.0020
MC difference of coef	1.8430	0.3430	0.0185	0.0656	0.0471
MC product of coef	1.8430	0.3430	0.0185	0.0418	0.0232

N=200	Avg $\widehat{\text{IDE}}$	Bias $\widehat{\text{IDE}}$	Var $\widehat{\text{IDE}}$	Avg $\widehat{\text{Var}}(\widehat{\text{IDE}})$	Bias $\widehat{\text{Var}}(\widehat{\text{IDE}})$
Regression formula	1.3788	-0.1212	0.0043	0.0043	-0.0001
Sobel's formula	1.3788	-0.1212	0.0043	0.0171	0.0128
Bootstrap cases	1.3773	-0.1227	0.0043	0.0175	0.0131
Bootstrap residuals	1.3787	-0.1213	0.0043	0.0042	-0.0001
MC difference of coef	1.3787	-0.1213	0.0043	0.0286	0.0242
MC product of coef	1.3787	-0.1213	0.0043	0.0172	0.0128

Table 1. Results of 5000 simulations (10,000 bootstrap and 10,000 Monte Carlo replications per simulation) of the estimated indirect effect and its estimated variance under the simple mediation model with a true indirect effect of 1.5 for sample sizes N = 50, 100, 200. Note that for the simple mediation model, the (natural) indirect effect for a unit change in the exposure equals the Essential Mediation Component Δ. We use $\widehat{\text{IDE}}$ as an acronym for the indirect effect.

Scenarios where PE = NIE	Causal Mediation Models*
Simple mediation model	$\mathbf{E}[\boldsymbol{Y}\vert\boldsymbol{X},\boldsymbol{M}] = \boldsymbol{\beta_0} + \boldsymbol{\beta_X}\boldsymbol{X} + \boldsymbol{\beta_M}\boldsymbol{M}$<br>$\mathrm{E}[M\vert X] = \alpha_0 + \alpha_X X$
Confounders	$\mathbf{E}[\boldsymbol{Y}\vert\boldsymbol{X},\boldsymbol{M},\boldsymbol{C}] = \boldsymbol{\beta_0} + \boldsymbol{\beta_X}\boldsymbol{X} + \boldsymbol{\beta_M}\boldsymbol{M} + \boldsymbol{\beta_C}\boldsymbol{C}$<br>$\mathrm{E}[M\vert X,C] = \alpha_0 + \alpha_X X + \alpha_C C$
Exposure-confounder interaction	$\mathbf{E}[\boldsymbol{Y}\vert\boldsymbol{X},\boldsymbol{M},\boldsymbol{C}] = \boldsymbol{\beta_0} + \boldsymbol{\beta_X}\boldsymbol{X} + \boldsymbol{\beta_M}\boldsymbol{M} + \boldsymbol{\beta_C}\boldsymbol{C} + \boldsymbol{\beta_{XC}}\boldsymbol{XC}$<br>$\mathrm{E}[M\vert X,C] = \alpha_0 + \alpha_X X + \alpha_C C + \alpha_{XC} XC$
Multiple mediators	$\mathbf{E}[\boldsymbol{Y}\vert\boldsymbol{X},\boldsymbol{M_1},\boldsymbol{M_2}] = \boldsymbol{\beta_0} + \boldsymbol{\beta_X}\boldsymbol{X} + \boldsymbol{\beta_{M_1}}\boldsymbol{M_1} + \boldsymbol{\beta_{M_2}}\boldsymbol{M_2}$<br>$\mathrm{E}[M_1\vert X] = \alpha_{01} + \alpha_1 X$<br>$\mathrm{E}[M_2\vert X] = \alpha_{02} + \alpha_2 X$
Multiple mediators with confounders	$\mathbf{E}[\boldsymbol{Y}\vert\boldsymbol{X},\boldsymbol{M_1},\boldsymbol{M_2},\boldsymbol{C}] = \boldsymbol{\beta_0} + \boldsymbol{\beta_X}\boldsymbol{X} + \boldsymbol{\beta_{M_1}}\boldsymbol{M_1} + \boldsymbol{\beta_{M_2}}\boldsymbol{M_2} + \boldsymbol{\beta_C}\boldsymbol{C}$<br>$\mathrm{E}[M_1\vert X,C] = \alpha_{01} + \alpha_1 X + \alpha_{C1} C$<br>$\mathrm{E}[M_2\vert X,C] = \alpha_{02} + \alpha_2 X + \alpha_{C2} C$
Mediator-mediator interactions	$\mathbf{E}[\boldsymbol{Y}\vert\boldsymbol{X},\boldsymbol{M_1},\boldsymbol{M_2}] = \boldsymbol{\beta_0} + \boldsymbol{\beta_X}\boldsymbol{X} + \boldsymbol{\beta_{M_1}}\boldsymbol{M_1} + \boldsymbol{\beta_{M_2}}\boldsymbol{M_2} + \boldsymbol{\beta_{M_1M_2}}\boldsymbol{M_1M_2}$<br>$\mathrm{E}[M_1\vert X] = \alpha_{01} + \alpha_1 X$<br>$\mathrm{E}[M_2\vert X] = \alpha_{02} + \alpha_2 X$<br>$\mathrm{E}[M_1M_2\vert X] = \alpha_{03} + \alpha_3 X$

* Bolded equation represents the fitted model used in the proposed framework

Table 2. Commonly encountered mediation models for exposure X, mediator M, outcome Y, confounders C where the portion eliminated (PE) and the natural indirect effect (NIE) are equal. The models used to estimate mediation effects in the traditional causal framework are shown in the second column. Using the proposed single model framework requires fitting only the first model listed under for each scenario, shown in bold. Unless otherwise specified, the exposure can be any type of variable and the mediator and outcome are continuous variables.

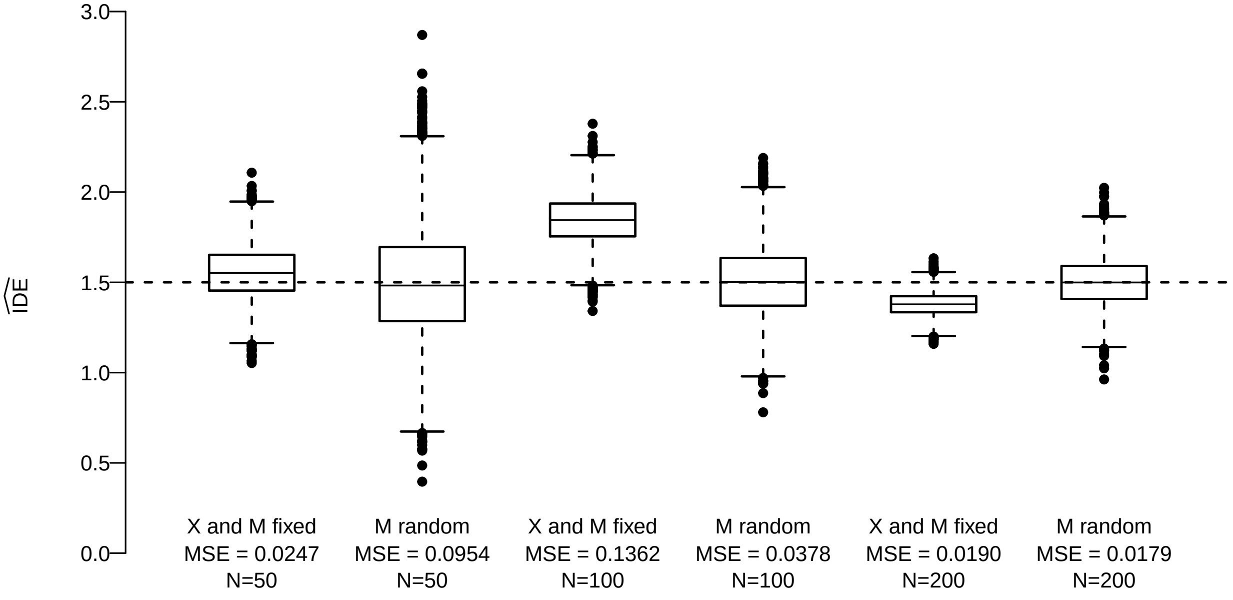

Fig. 2. We use $\widehat{\text{IDE}}$ as an acronym for the estimated (natural) indirect effect. Results of 5000 simulations of $\widehat{\text{IDE}}$ from the simple mediation model for sample sizes $N = 50, 100, 200$. Note that using the product of coefficients or difference of coefficients yields equivalent estimates of $\widehat{\text{IDE}}$ for this simple model. We compare the effect of treating both X and M as fixed covariates (i.e. at every simulation a new Y is generated conditional on the same X and M) and treating X as fixed and M as a random variable (i.e. at every simulation a new M and a new Y are generated). Notice that when M is treated as a random variable, the bias of $\widehat{\text{IDE}}$ is reduced. Intuitively, when we let M vary, r_{XM} varies and better approximates ρ_{XM} than the sample correlation from a single draw from the distribution of M. We notice the bias-variance tradeoff since when M is random we also see an increased variance in the distribution of $\widehat{\text{IDE}}$. For N = 50, $\text{cov}(x, m) = 2.606$, $\text{var}(x) = 2.514$, $\text{var}(m) = 6.740$. For N = 100, $\text{cov}(x, m) = 4.785$, $\text{var}(x) = 4.156$, $\text{var}(m) = 9.316$. For N = 200, $\text{cov}(x, m) = 3.227$, $\text{var}(x) = 3.510$, $\text{var}(m) = 6.933$.
